# Impact of gut microbial diversity on egg production performance in chickens

**DOI:** 10.1128/spectrum.01927-24

**Published:** 2025-01-14

**Authors:** Liping Wu, Tao Zhang, Zhihua Luo, Huiyuan Xiao, Di Wang, Cailong Wu, Xinyan Fang, Jiawei Li, Jing Zhou, Junjie Miao, Hongli Tan, Yanan Wang, Qing Liu, Jianhua Huang

**Affiliations:** 1 College of Life Science, Jiangxi Science and Technology Normal University, Nanchang, China; 2 Forestry Bureau of Yushui District, Xinyu City, Nanchang, China; 3 Jiangxi Key Laboratory of Natural Microbial Medicine Research, Nanchang, China; 4 Tonggu County, Jiangxi Province Agriculture Rural Water Resources Bureau, Yichun, China; 5 Jinxian Hengrong Ecological Agriculture Development Co. Ltd., Nanchang, China; Lerner Research Institute, Cleveland, Ohio, USA

**Keywords:** chickens, egg production, intestinal microbiota, 16S rRNA sequencing

## Abstract

Chickens are one of the most economically important poultry species, and their egg-laying performance is a crucial economic trait. The intestinal microbiome plays a significant role in the egg-laying performance. To clarify the diversity of chicken intestinal microbiota and its connection to egg-laying performance, this study utilized 16S rRNA sequencing technology to characterize the intestinal microbiomes of 101 chickens from 13 breeds with varying levels of egg production. The results reveal significant differences in gut microbiota structure among chicken groups with varying egg production levels. High egg-producing chickens showed significantly higher abundances of *Firmicutes*, *Proteobacteria*, and *Lactobacillus*, while low egg-producing chickens displayed greater microbial α-diversity and more complex community structures. These differences in gut microbiota influence key physiological functions, including nutrient absorption and hormone regulation through metabolic pathways, and directly affect egg production performance. The low and medium production groups partially overlapped on the principal coordinates analysis plot, whereas the high-production group was distinctly separate. This study provides a scientific basis and intestinal microbiome data for selecting probiotics related to high egg production in chickens.

**IMPORTANCE:**

This study elucidates the critical role of gut microbiota in the egg-laying performance of chickens, a key economic indicator in the poultry industry. By employing 16S rRNA sequencing, we uncovered distinct microbial profiles associated with varying levels of egg production. High egg-producing chickens exhibit a higher abundance of specific bacterial taxa, such as *Firmicutes* and *Proteobacteria*, which are linked to enhanced nutrient absorption and metabolic efficiency. Conversely, lower and medium egg-producing chickens display greater microbial diversity, suggesting a more complex but less efficient gut ecosystem. Our findings provide valuable insights into the relationship between gut microbiota and egg production, offering a scientific foundation for the selection of probiotics that could potentially improve the egg-laying performance of chickens. This research not only advances our understanding of avian gut microbiology but also has practical implications for optimizing poultry farming practices and enhancing economic outcomes.

## INTRODUCTION

Chickens, with their outstanding production performance and economic benefits, have become an indispensable and significant part of the livestock and poultry farming industry (1). Examples of economically important chicken breeds include the Ning du San huang chicken (ND) (2), Taihe Silkie chicken (TH) (3), and Hailan Brown chicken (HLB) (4). They are all highly esteemed breeds, each with unique characteristics and egg-laying capabilities. They adapt well to local climates and environments, providing delicious, nutritious meat. During the breeding process, these chickens can adapt well to natural environments, effectively increasing farmers’ income. High-yield egg-laying chickens exhibit strong resilience and unique egg-laying traits. A deeper understanding of the composition and function of the local chicken gut microbiome can help optimize feeding and management strategies, enhancing the egg-laying performance of local chickens. Currently, research by Kazemi and others indicates that gut microbiota influence the growth performance of chickens (5), Additionally, research by Abd El-Hack and others has shown that gut microbiota influence the immune system (6). However, our understanding of how gut microbiota influences egg-laying performance is still insufficient.

Egg-laying performance is one of the most important economic traits in chickens, and consumer preference for traditional breed egg products is on the rise. This is crucial for achieving efficient poultry farming and meeting market demand for eggs (7). For chickens, laying eggs is an important process for reproducing offspring (8). Egg laying is not just a physiological activity; it is also essential for the propagation of chicken populations. Research has identified multiple factors that can influence egg-laying performance (9). Research indicates that there are distinct differences in the gut microbial profiles between high-producing (H) and low-producing (L) hens (10). Studies also show that variations in feed composition and hormonal changes influence these microbial communities. Analysis of 16S rDNA sequencing and fecal microbiota transplantation reveals (11, 12) that *Firmicutes* dominate both groups. However, there is a greater abundance of *Bacteroidetes*, *Actinobacteria*, and *Proteobacteria* in low-producing hens (10). When gut microbiota from high-producing hens were transplanted into low-producing hens, their egg-laying rate significantly improved, while the reverse transplantation caused only a short-term fluctuation (10). Further research demonstrates that changes in the composition of gut microbiota are caused throughout the rearing period and continue until the end of the laying cycle (around 80 weeks of age) (13), for example, *Lactobacillus* predominates in the upper gastrointestinal tract of chicks, but it mainly colonizes the lower gastrointestinal tract of adult hens (13). Additionally, researchers observed a higher proportion of *Proteobacteria*, including opportunistic pathogens like Gallibacterium, with their relative abundance increasing after the peak of egg production (13).

A healthy gut microbial community helps improve the nutrient absorption and utilization efficiency in chickens, for example, dietary fiber can be used to regulate the composition of the gut microbiome (14), thereby promoting chicken growth and egg-laying performance. Smith et al. reported that adding alfalfa meal to chicken feed can positively affect production performance, potentially enhancing egg-laying capabilities and increasing the diversity of the gut microbiome (15). Research has found that the gut microbiome can be improved through diet (16). Additionally, the microbiota in the gut is not static but in a dynamic equilibrium. Disruption of this balance can lead to metabolic disorders in the host and adversely affect egg-laying performance (17). However, maintaining the normal physiological functions of the gut microbiome can improve egg-laying performance. For example, the diversity of the bacterial communities in the chicken gut can be altered by adding supplements such as nutrients in health products (probiotics, prebiotics, etc.) (18) and herbs (polyphenols, herbal remedies, spices, etc.) (19). The aim of this study is to explore how to regulate the gut microbiome of chickens, maintain its dynamic balance, and enhance egg-laying performance and economic outcomes.

To elucidate the relationship between the gut microbiota of chickens and their egg production, we utilized 16S rRNA sequencing to categorize chickens of different breeds into three groups based on egg production levels: low, medium, and high. Our research explores the diversity and abundance of gut microbiota in chickens, identifying significant differences among groups with varying egg production levels and offering insights for improving egg-laying performance.

## RESULTS

### Evaluation of sequencing data

**Fig 1:**
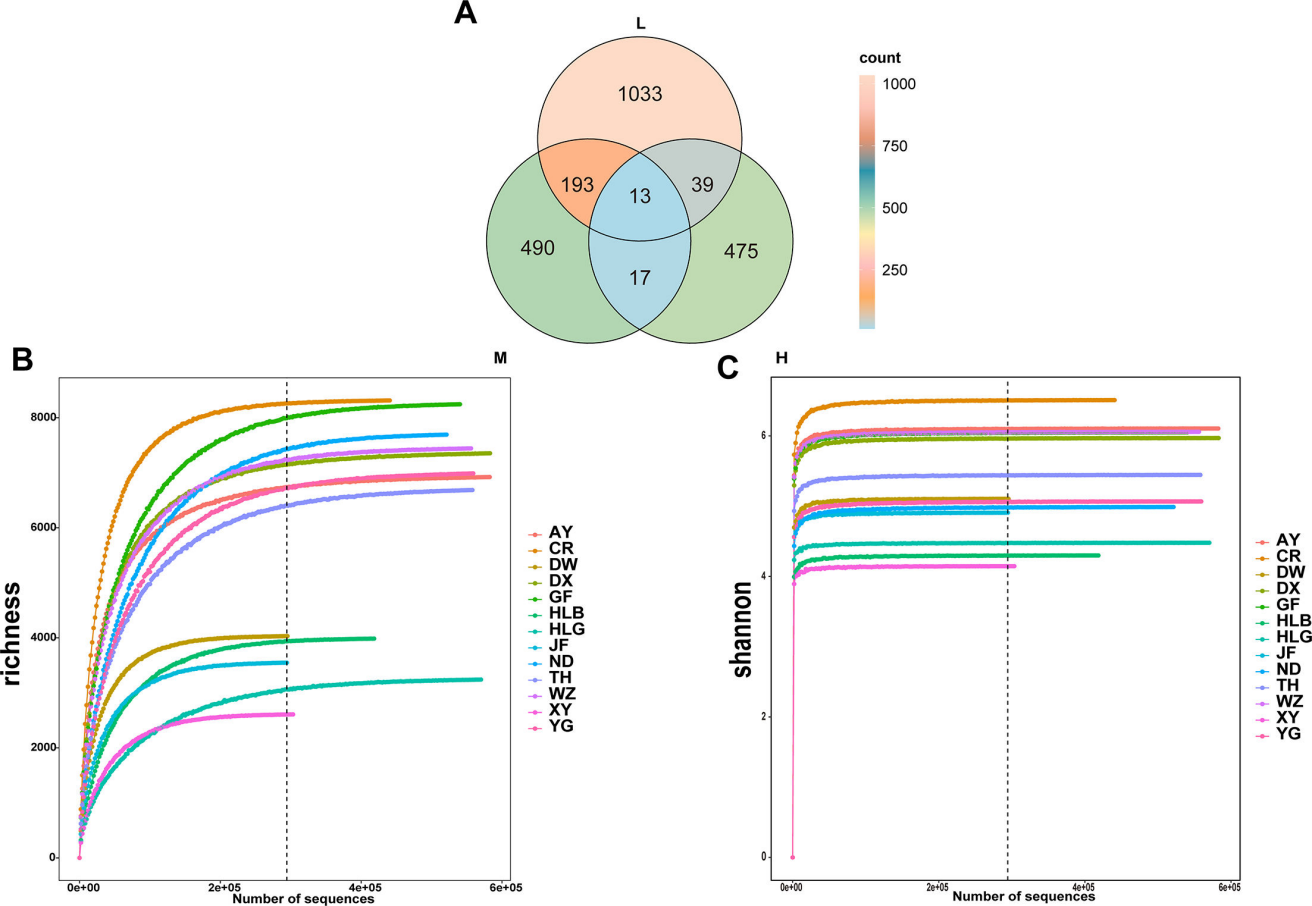
Overview of sequencing data. (**A**) Venn diagram of sample grouping; (**B**) rarefaction curve of sample grouping; (**C**) Shannon curve of sample grouping. L indicates low-yield group, M indicates medium-yield group, H indicates high-yield group, 0e+00 indicates no value or missing value, 2e+05 indicates $2\times 10^{5}$, 4e+05 indicates $4\times 10^{5}$, and 6e+05 indicates $6\times 10^{5}$.

To elucidate the composition of the fecal microbiota communities in chickens with different egg-laying capacities, fecal samples from three groups of chickens with varying egg production performances were analyzed. After sequencing 101 samples, the results are shown in Table 1. The results are as follows: among these three groups, the low egg-producing group had the highest number of unique operational taxonomic units (OTUs), indicating a more diverse gut microbiota. This higher microbial diversity may represent an adaptation to a more variable environment. Low egg-producing chickens shared 193 OTUs with medium egg-producing chickens, while medium egg-producing chickens shared 17 OTUs with high egg-producing chickens, and low egg-producing chickens shared 39 OTUs with high egg-producing chickens. The highest number of shared OTUs was between low and medium egg-producing chickens, with all three groups sharing 13 OTUs. These shared OTUs are important components of the bacterial community structure and indicate a correlation between egg production levels and the diversity of the gut microbiome, as shown in Fig. 1A. The Shannon curves and rarefaction curves show that the number of sequences is sufficient to represent each microbial community adequately, ensuring the analysis’s reliability (as shown in Fig. 1B and C). The rank abundance curves demonstrate high species richness and even distribution, suggesting the sample size is adequate and reasonable for this experiment (Fig. S1).

**TABLE 1:** Sample sequencing data analysis*^a^*^,^*^b^*

Egg production group	Group	Clean reads	ACE	OTUs	Shannon	Coverage (%)
Low egg-producing chickens	AY	639,257	1,071.51 ± 293.72	1,054	6.94 ± 1.04	99.94%
	DX	638,026	1,225.11 ± 560.05	1,212	6.86 ± 1.95	99.94%
	TH	683,587	1,097.99 ± 486.38	1,082	6.02 ± 1.91	99.93%
	WZ	638,574	1,272.54 ± 293.72	1,263	7.11 ± 2.25	99.95%
	YG	639,029	1,145.86 ± 255.28	1,136	6.07 ± 1.14	99.94%
Medium egg-producing chickens	ND	695,544	1,116.31 ± 590.05	1,104	5.63 ± 1.88	99.94%
	CR	478,704	1,816.48 ± 327.98	1,812	8.10 ± 1.85	99.97%
	GF	638,823	1,302.57 ± 409.49	1,292	6.95 ± 1.57	99.94%
High egg-producing chickens	DW	331,315	767.4433 ± 256.61	764	5.65 ± 1.03	99.96%
	HLB	486,135	509.49 ± 279.95	505	4.63 ± 0.82	99.96%
	HLG	668,012	359.01 ± 210.60	353	4.90 ± 0.98	99.96%
	JF	329,885	683.81 ± 251.62	680	5.70 ± 1.28	99.96%
	XY	347,495	484.88 ± 162.76	482	4.54 ± 1.32	99.97%

^*a*^ Total raw reads: the total number of raw sequences obtained from samples using high-throughput sequencing technology. Total clear reads: the number of high-quality sequences remaining after quality filtering of the raw sequences, suitable for further analysis. Minimum clean reads/sample: the minimum number of clean sequences obtained in a single sample across all samples. Average clean reads/sample: the average number of clean sequences obtained after quality filtering for each sample across all samples. Total OTUs: the total number of OTUs identified across all samples. Average OTUs/sample: the average number of OTUs identified in each sample, reflecting the microbial diversity of each sample. ACE = abundance-based coverage estimator.

^*b*^ Abbreviations: AY, Anxi Gray; DX, Dongxiang Blue eggshell; TH, Taihe Silkie; WZ, Wanzai Kangle; YG, Yugan Black; CR, Chongren Partridge; GF, Guangfeng Baier; ND, Ningdu Yellow; DW, Dawu Golden Phoenix; HLG, Hailan Grey; HLB, Hailan Brown; JF, Jinfen No. 6; XY, Xinyang Black. N represents the number of samples; average egg production is shown as the number of eggs.

### Analysis of intestinal microbial diversity in chickens with different laying performance

**Fig 2:**
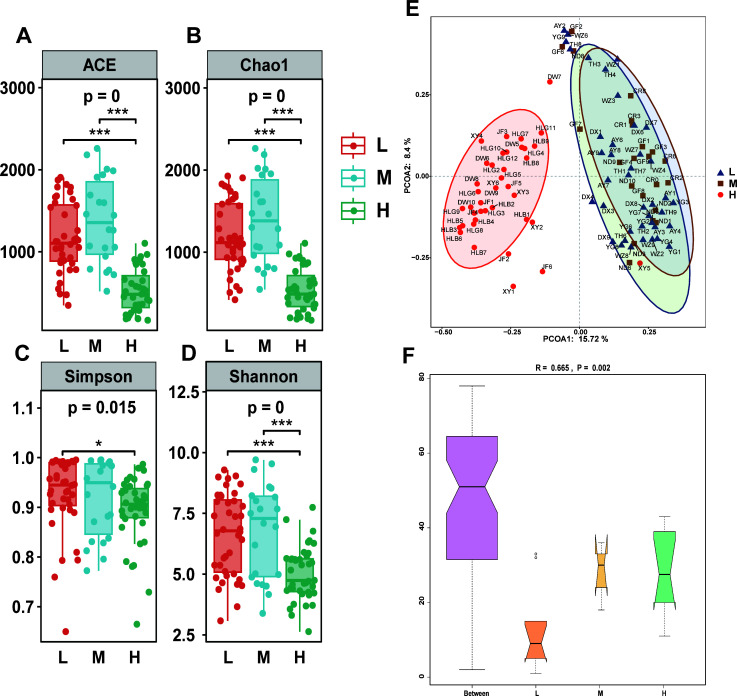
Microbial diversity analysis by different egg production groups. (**A**) ACE index analysis; (**B**) Chao1 index analysis; (**C**) Simpson index analysis; (**D**) Shannon index analysis; (**E**) principal coordinates analysis (PCoA) plot of different species based on Bray-Curtis distance; (**F**) Analysis of Similarities (ANOSIM) Plot. Caption P=0 indicates significant differences; *P<0.05, **P<0.01, and ***P<0.001.

To explore the gut microbial diversity among chickens with different egg-laying capacities, various alpha diversity indices were evaluated based on the OTU abundance, as shown in Table 2. The ACE and Chao results indicate that there are significant differences in the richness of gut microbial OTUs among groups with different egg production levels (as shown in Fig. 2A and B). The results indicate that the richness of gut microbial OTUs in the low and medium egg-producing chickens is greater than that in high egg-producing chickens. More importantly, across various alpha diversity indices, the diversity and species richness of the gut microbiota in low and medium egg-producing chickens are higher than those in high-producing chickens (as shown in Fig. 2C and D). Additionally, as shown in Table 2, the coverage of the text is 99.94% and above, which is quite high. This suggests a high probability that the species in the sample have been detected, and a low probability that any have been missed. This index reflects that the sequencing results likely represent the true situation of the microbiome in the samples (Fig. S2).

**TABLE 2:** Quality description for each group^*a*^

Egg production group	Group	Clean reads	ACE	OTUs	Shannon	Coverage (%)
Low egg-producing chickens	AY	639,257	1,071.51 ± 293.72	1,054	6.94 ± 1.04	99.94%
	DX	638,026	1,225.11 ± 560.05	1,212	6.86 ± 1.95	99.94%
	TH	683,587	1,097.99 ± 486.38	1,082	6.02 ± 1.91	99.93%
	WZ	638,574	1,272.54 ± 293.72	1,263	7.11 ± 2.25	99.95%
	YG	639,029	1,145.86 ± 255.28	1,136	6.07 ± 1.14	99.94%
Medium egg-producing chickens	ND	695,544	1,116.31 ± 590.05	1,104	5.63 ± 1.88	99.94%
	CR	478,704	1,816.48 ± 327.98	1,812	8.10 ± 1.85	99.97%
	GF	638,823	1,302.57 ± 409.49	1,292	6.95 ± 1.57	99.94%
High egg-producing chickens	DW	331,315	767.4433 ± 256.61	764	5.65 ± 1.03	99.96%
	HLB	486,135	509.49 ± 279.95	505	4.63 ± 0.82	99.96%
	HLG	668,012	359.01 ± 210.60	353	4.90 ± 0.98	99.96%
	JF	329,885	683.81 ± 251.62	680	5.70 ± 1.28	99.96%
	XY	347,495	484.88 ± 162.76	482	4.54 ± 1.32	99.97%

^*a*^ Clean reads refer to the high-quality reads obtained after quality control of the raw sequences. ACE (abundance-based coverage estimator) is an index used to estimate the number of species in a community based on abundance; Shannon index (Shannon–Wiener index) is one of the indices used to estimate microbial diversity in samples; coverage refers to the coverage rate of the sample library.

In the principal coordinates analysis (PCoA) plot (as shown in Fig. 2E), different colors represent different groups, and each scatter point represents an individual. The PCoA plot reveals a clear distinction between the high egg-producing group and other groups. The microbial communities of the high egg-producing group are distinctly separated from those of the low and medium egg-producing groups, while there is no significant separation between the low and medium egg-producing groups. Additionally, this study also conducted ANOSIM analysis, which tested the similarity of gut microbiota community structures among low, medium, and high egg-producing chickens, as well as among different chicken breeds. In conclusion, the results clearly indicate that there are highly significant differences in the gut microbiota structures among the three different egg-producing chicken groups. These findings underscore the biostatistical significance of the groupings in this experimental design (*R* = 0.665, *P* = 0.002), as shown in Table 3 (as shown in Fig. 2F; Fig. S3).

**TABLE 3:** Analysis of differences in the gut microbiota community structure of three groups of chickens using the ANOSIM method^*a*^

Group	Distance	*R*	*P* value	OTUs	Shannon	Coverage (%)
L/M	Bray-Curtis	0.107692	0.328	1,054	6.94 ± 1.04	99.94%
L/H	Bray-Curtis	0.964	0.008	1,212	6.86 ± 1.95	99.94%
M/H	Bray-Curtis	0.958974	0.013	1,082	6.02 ± 1.91	99.93%

^*a*^ L indicates low-yield group, M indicates medium-yield group, H indicates high-yield group, *R* indicates degree of difference, and *P* value = *p*-value.

### Differences and composition of gut microbiome in chickens with varying egg-laying performances

To understand the differences and composition of gut microbiomes among chicken samples with varying egg-laying performances, the top 10 bacterial phyla and genera in terms of abundance were displayed and analyzed. *Firmicutes*, *Actinobacteriota*, *Bacteroidota*, and *Proteobacteria* are the dominant bacterial phyla in the gut microbiome of local chicken breeds, cumulatively accounting for over 90% of all sequence relative abundances and forming the main characteristics of the chicken gut microbiome. Other phyla with lower relative abundances represent the broad taxonomic composition of the microbiome. At the microbial genus level, *Lactobacillus*, *Romboutsia*, and *Enterococcus* were the most common genus.

**Fig 3:**
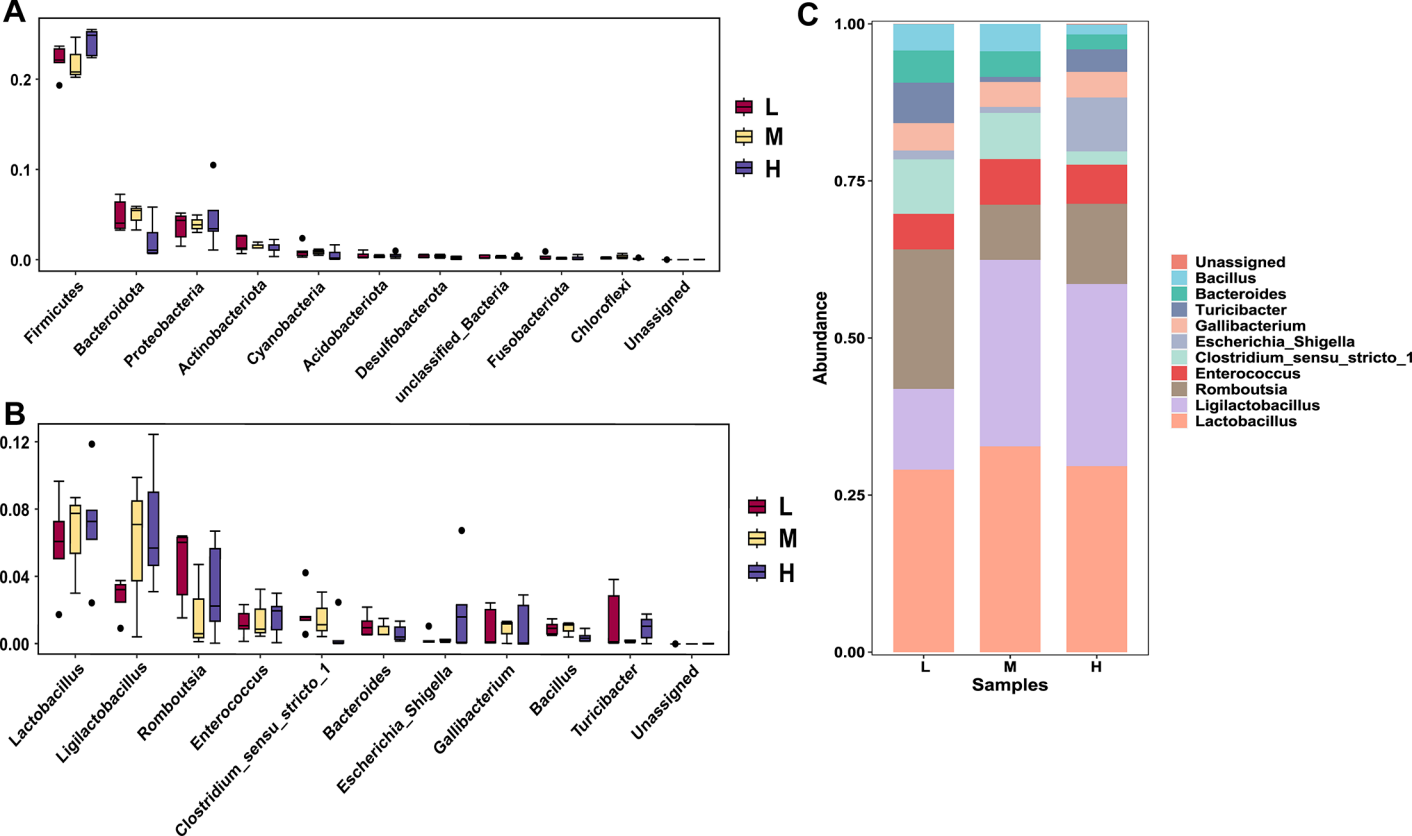
Top 10 microbial compositions and comparisons in different egg production groups. (**A**) Comparison of top 10 microbial differences by egg production groups; (**B**) comparison of gut microbiota at the genus level among different egg production groups; (**C**) histogram of species abundance at the genus level in different egg production groups.

This study compared the differences in abundance at the phylum and genus levels of gut microbiota among chickens with varying egg production levels. At the phylum level, it was found that high egg-producing chickens had a higher proportion of *Firmicutes* and *Proteobacteria* in their guts compared with low and medium egg-producing chickens, while the proportions of *Actinobacteriota* and *Bacteroidota* were greater in low and medium egg-producing chickens than in high egg-producing chickens (as shown in Fig. 3A; Fig. S4; Table S1).

The top 10 genera in terms of relative abundance were selected for analysis. In comparisons across the three groups of chickens with different egg production levels, it was observed that the high egg-producing group had the highest relative abundance of the genus *Lactobacillus*. The relative abundance of *Romboutsia* bacteria is highest in the group with low egg production. The relative abundance of *Ligilactobacillus* in the low-egg production group is the lowest. The relative abundance of *Enterococcus* is consistently present across low-, medium-, and high-egg production groups in 13 different breeds of chickens, with no significant differences observed; however, the concentration is highest in the high-egg production group. In summary, there were significant differences in gut microbiota abundance among different chicken groups, and these differences might affect egg production performance by influencing gut health, nutrient absorption efficiency, and metabolic functions. For example, the higher abundance of *Firmicutes* and *Lactobacillus* in the gut of high egg-producing chickens enhanced nutrient absorption efficiency and energy metabolism, resulting in higher egg production. Additionally, the increased abundance of *Lactobacillus* and *Enterococcus* might promote gut health, thereby improving the overall production performance of chickens (as shown in Fig. 3B and C; Table S2).

### Identification and functional prediction of biomarkers in chickens with different egg-laying performances

**Fig 4:**
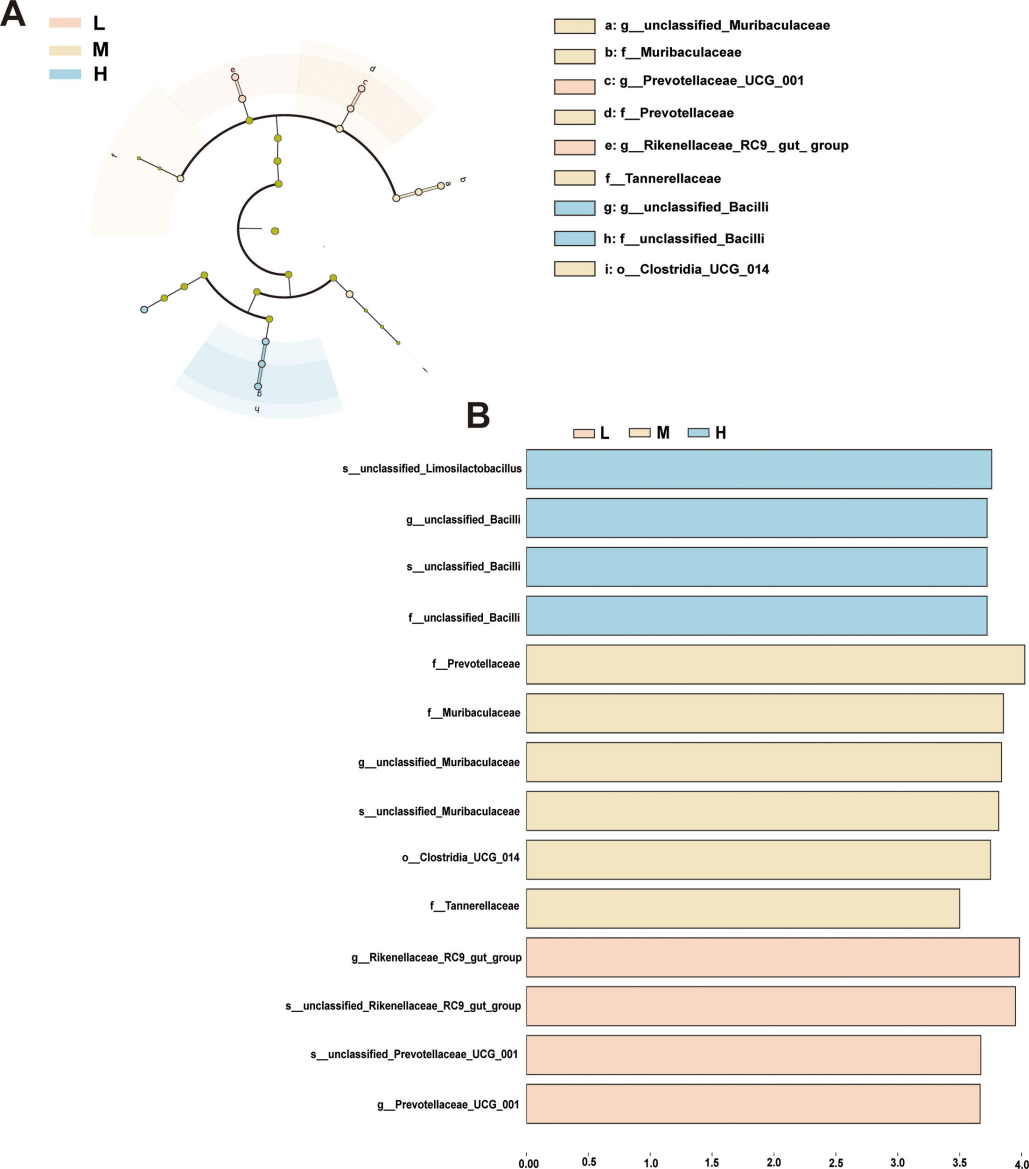
LEfSe analysis of gut microbiota by egg production. (**A**) Branch diagram of bacterial species at different classification levels; (**B**) LEfSe analysis among different varieties, with biomarkers at LDA threshold ≥3.5. The evolutionary branch diagram shows varying abundances from phylum to genus level. L indicates low-yield group, M indicates medium-yield group, and H indicates high-yield group.

There are clear differences in microbial species between groups at different taxonomic levels, indicating significant evolutionary differences. These differences may reflect their diversity and variation in egg production levels, genetics, and functionality. In this study, chickens were divided into three groups: L group (low-egg production group), M group (medium-egg production group), and H group (high-egg production group). We conducted an analysis of the gut microbial communities of chickens with varying egg production levels and used Linear Discriminant Analysis Effect Size (LEfSe) to identify microbial taxa, which were statistically significant and biologically stable among the groups. The low-egg production group has two biomarkers, while the medium egg production group has the most, with five biomarkers. The high-egg production group has two biomarkers (as shown in Fig. 4A). Through LEfSe analysis, specific clades with consistent changes in abundance can be identified in chickens with different egg-laying performances, which can serve as biomarkers. With the linear discriminant analysis (LDA) threshold set at 3.5, 14 specific biomarkers were identified among the L, M, and H groups (as shown in Fig. 4B). The results suggest that the number and types of biomarkers vary among chickens with different levels of egg production, indicating that these biomarkers are related to the production performance of the chickens. Specifically, these biomarkers may be involved in regulating or influencing the physiological processes of egg production. Among the low-, medium-, and high-egg production groups, the main biomarkers in group L are *g_Rikenellaceae_RC9_gut_group* and *g_Prevotellaceae_UCG_001*, both of which belong to the phylum Bacteroidetes.

The main biomarkers in group M are the families *f_Prevotellaceae*, *f_Muribaculaceae*, and *f_Tannerellaceae*. The main types of biomarkers in group H are from the *unclassified_Limosilactobacillus* family and the *unclassified_Bacilli* genus. Additionally, an LEfSe analysis with an LDA score >3 was performed, identifying 84 specific biomarkers across the L, M, and H groups. The low-egg production group has 20 biomarkers, while the medium-egg production group has the most, with 58 biomarkers. The main biomarkers of the high-egg production group are as previously mentioned (Fig. S5). The high-egg production group has the fewest, with six biomarkers (Fig. S6).

**Fig 5:**
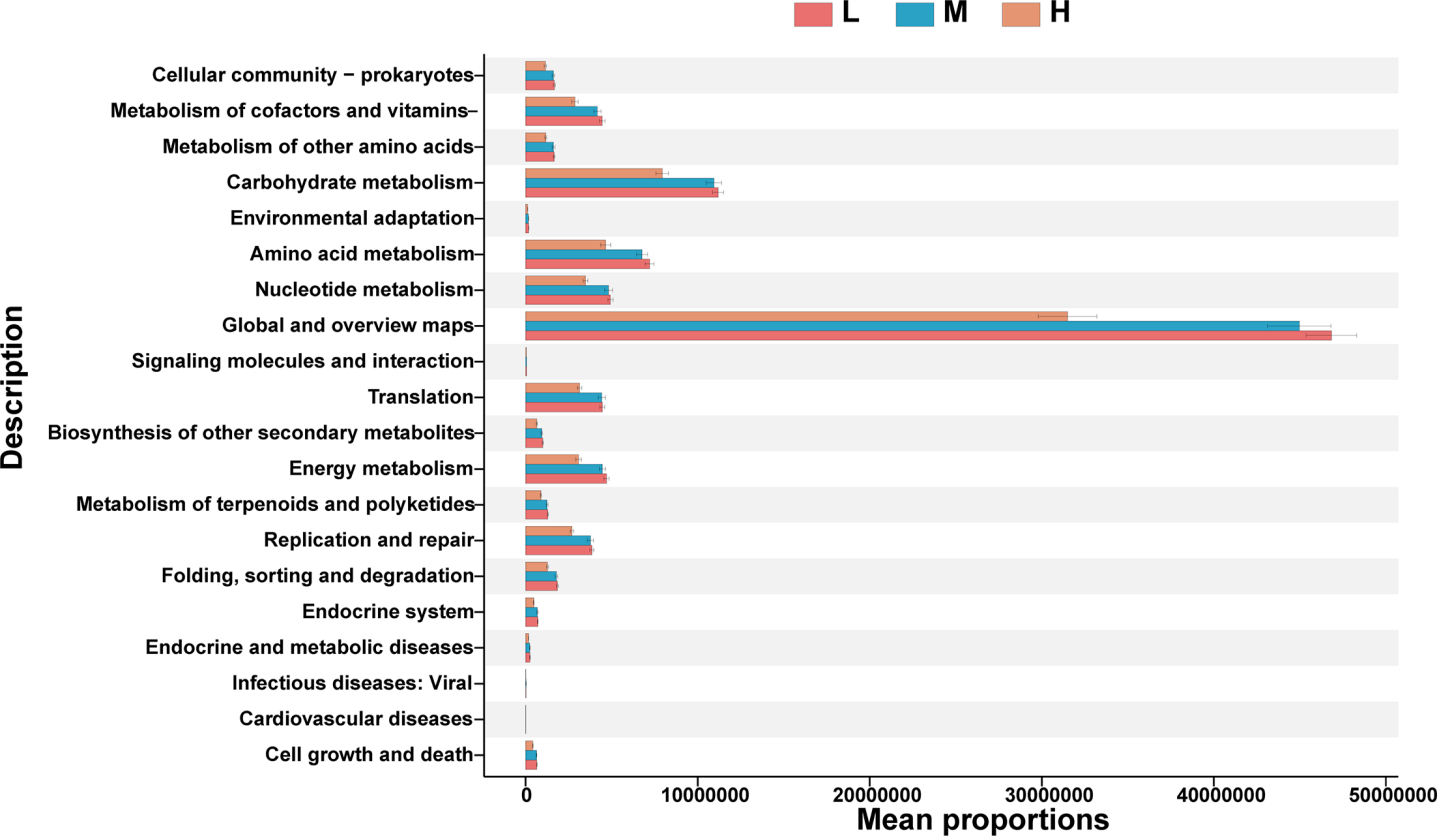
Differential analysis of KEGG secondary metabolic pathways.

Using STAMP software for differential analysis of Kyoto Encyclopedia of Genes and Genomes (KEGG) level 2 metabolic pathways, 46 functional categories were identified. Comparing the functional differences among the three groups with different egg production levels, 20 level 2 functional metabolic pathways showed significant differences. The gut microbiota of chickens in the low-egg production group are primarily involved in amino acid metabolism, energy metabolism, folding, sorting and degradation, nucleotide metabolism, and translation. This suggests that the microbial communities in the low-egg production group are more involved in basic biochemical processes and in maintaining fundamental biological activities. The gut microbiota of chickens in the medium-egg production group are primarily involved in cellular communities: prokaryotes, metabolism of other amino acids, biosynthesis of other secondary metabolites, metabolism of terpenoids and polyketides, the endocrine system, and cell growth and death. These functions involve biosynthesis and complex metabolic activities, which may help maintain the health and balance of the gut microbial community, thereby indirectly supporting as table egg production level. The regulation of cell growth and death may also be key to adjusting the internal environment to meet production demands. The gut microbiota of chickens in the high-egg production group are primarily involved in the metabolism of cofactors and vitamins, carbohydrate metabolism, endocrine and metabolic diseases, infectious diseases: viral, and cardiovascular diseases. In summary, these results indicate that there are functional differences in the gut microbiota of chickens among different egg production groups. The functional characteristics of the chicken gut microbiota can, to some extent, explain the differences between the different egg production groups (as shown in Fig. 5). Additionally, the results of KEGG level 3 metabolic pathways also show significant differences among the different egg production groups (Fig. S7).

## MATERIALS AND METHODS

### Sample collection

**Fig 6:**
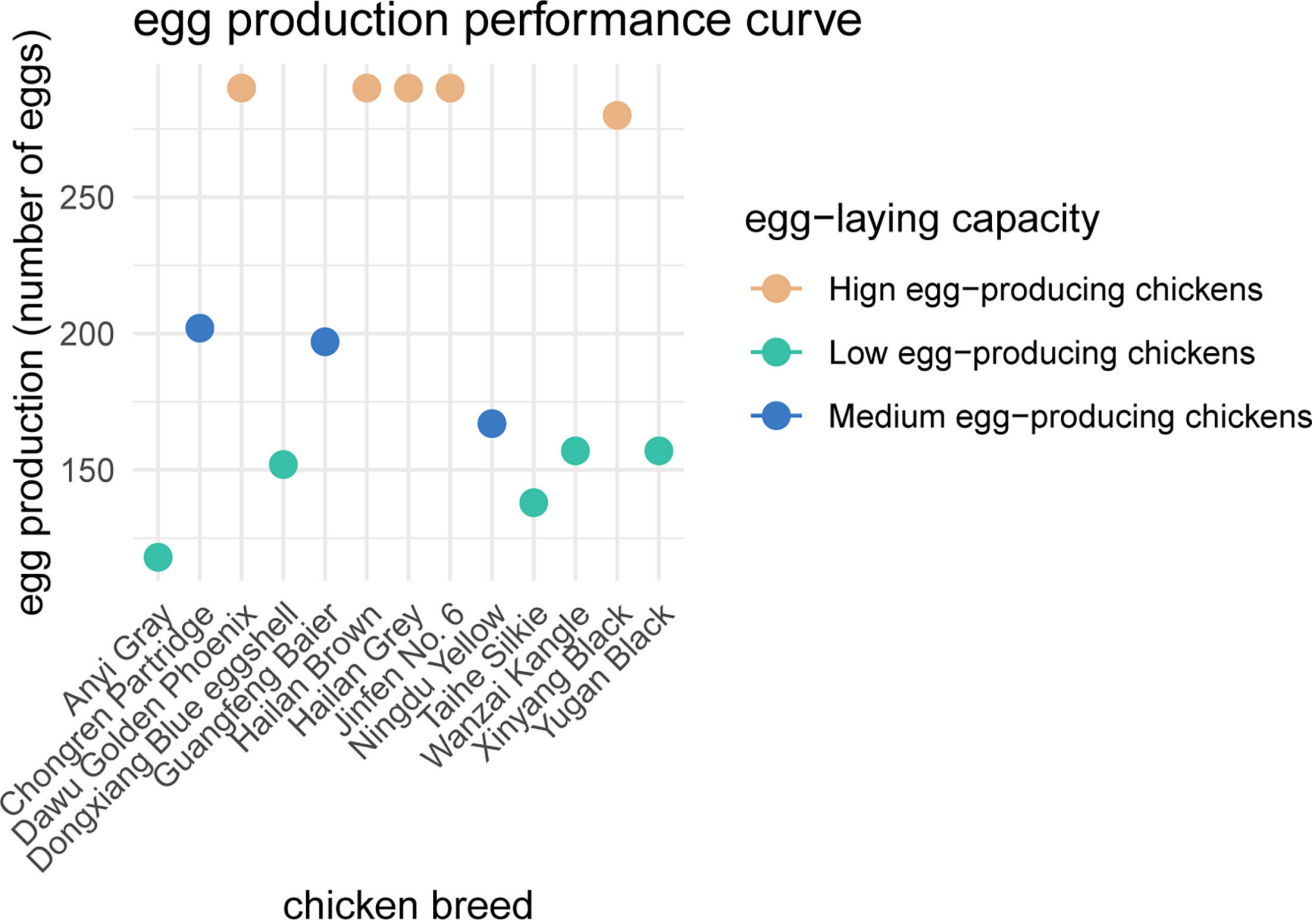
Egg production performance curve. The curve illustrates the trend of egg production over time, showing the average production levels across the different groups (L indicates low yield, M indicates medium yield, and H indicates high yield) throughout the 500-day period.

We collected fecal samples from 101 chickens across 13 breeds at a poultry farm and categorized these individuals into three groups based on their egg production levels: low, medium, and high, as shown in (Table 4; Fig. 6). In this study, the rearing environment for the chickens strictly adhered to the feeding standards set by the National Research Council of the United States. The diet for all chickens was based on a corn-soybean meal formulation. The temperature in the rearing environment was maintained at 24-28°C. During the feeding process, experimental chickens were collected from four different farms to ensure data diversity and representativeness. Different breeds of chickens were housed in separate cages to avoid interference between breeds, while chickens of the same breed were grouped with seven individuals per cage. This rearing method helps reduce overcrowding and stress, thereby improving the reliability and repeatability of experimental results. To ensure that the chickens received sufficient light, artificial lighting was used throughout the rearing process, with white light illumination provided for 16 hours a day, followed by 8 hours of darkness. The light intensity was set at 25 lux to maintain their health and production performance. Through these stringent control measures, this study ensured a uniform living environment for the experimental chickens, minimizing external environmental interference with the experimental results.

**TABLE 4:** Egg production characteristics of low-, medium-, and high-yield groups^*a*^

Egg-laying performance	Chicken breeds	Sample collection site	Samples size (*n*)	500 days of age (the number of eggs)	Adult body weight (kg)	Age of laying (days)	Egg weight (g)
Low egg-producing chickens	Anyi Gray (AY)	Nanchang City	8	103–133	1.447 ± 0.177 (F), 1.739 ± 0.242 (M)	145–155	45 ± 3.6
	Dongxiang Blue eggshell (DX)	Yingtan City	8	152	1.350 ± 0.183 (F), 1.719 ± 0.149 (M)	170–180	48.1 ± 3.7
	Taihe Silkie (TH)	Ji ’an City	8	138	1.379 ± 0.221 (F), 1.763 ± 0.173 (M)	156	43.6 ± 4.1
	Wanzai Kangle (WZ)	Pingxiang City	8	157	1.389 ± 0.143 (F), 1.801 ± 0.163 (M)	119–170	53.7 ± 4.6
	Yugan Black (YG)	Jingdezhen City	8	157	1.363 ± 0.221 (F), 1.776 ± 0.167 (M)	156	42 ± 3.6
Medium egg-producing chickens	Chongren Partridge (CR)	Fuzhou City	6	202	1.138 ± 0.115 (F), 1.629 ± 0.160 (M)	154–161	43.4 ± 3.4
	Guangfeng Baier (GF)	Shangrao City	8	197	1.174 ± 0.188 (F), 1.420 ± 0.092 (M)	147–152	54.1 ± 3.7
	Ningdu Yellow (ND)	Ganzhou City	8	167	1.300 ± 0.132 (F), 2.135 ± 0.072 (M)	135	50 ± 3.3
High egg-producing chickens	Dawu Golden Phoenix (DW)	Ganzhou City	6	280–300	1.8	130	61.11
	Hailan Grey (HLG)	Jinxian County	12	280–300	1.55–-2.05	130	63.19
	Hailan Brown (HLB)	Jinxian County	9	280–300	About 2.05	130	61.81
	Jinfen No.6 (JF)	Ganzhou City	6	280–300	1.8	130	61.11
	Xinyang Black (XY)	Anyi County	6	260–280	1.8	125	59.72

^*a*^ Note. *n* represents the sample size.

### Fecal DNA extraction and 16S rRNA gene amplicon sequencing

Total genomic DNA was extracted from the fecal samples of each chicken. Specific primers were synthesized from the conserved regions of the sequence, with the forward primer being (338F: 5′-ACTCCTACGGGAGGCAGCA-3′) and the reverse primer being (806R: 5′-GGACTACHVGGGTWTCTAAT-3′). Additionally, Illumina adapters were attached to the ends of the primers for PCR amplification. The PCR products were then recovered, purified, quantified, and homogenized to prepare the amplicon library for sequencing. Before sequencing, the constructed library was checked using the Qsep-400 method. Finally, the library was sequenced using the Illumina NovaSeq 6000 system, producing paired-end reads, also known as raw data.

### Data analysis

To ensure more accurate and reliable results, the raw data were first quality filtered using Trimmomatic (20). Subsequently, the Cutadapt software (21) was used to identify and remove sequences, generating clean reads. Next, USEARCH (version10) (22) was employed to assemble reads from each sample and remove chimeras. Chimera detection and removal were performed using UCHIME (version 8.1) (23). This algorithm aligned the tags with a reference database (Silva database Release 138, http://www.arb-silva.de) to eliminate chimera sequences and obtain valid tags. Ultimately, this process yielded high-quality, valid sequences.

### Sample classification and statistical analysis

To understand the characteristics of the chicken gut microbiota community, the valid sequences were obtained using Usearch (24). The sequences obtained from the software were clustered into the same OTUs at 97% similarity. The results of the classified OTUs allow for an analysis of the diversity of the samples. Additionally, the QIIME2 (25) software can be used to assess the community composition of each sample, producing species abundance tables at various taxonomic levels. Venn diagrams are employed to identify species that are unique or shared between different sample types (26). Alpha diversity indices, such as Shannon and ACE index, are utilized to analyze species diversity and complexity within the samples. Subsequently, an appropriate method based on the OTU level is used to generate a PCoA (27), which helps visualize the relationships among samples. Due to the complexity and diversity of the gut microbiome being significantly influenced by external environments, non-metric multidimensional scaling (ANOSIM) can also be applied to assess the difference sin species complexity between samples (28).

### Identification of biomarkers and functional prediction

LEfSe (29) is employed to identify significant biomarkers differentiating groups within the samples. The analysis begins with using the Kruskal-Wallis rank test (30) to detect differences in species abundance between groups. This is followed by the Wilcoxon rank-sum test (31) to determine if all subspecies of significant species trend toward the same taxonomic level. Finally, based on LDA, the definitive significant biomarkers are identified. PICRUSt2 (32) is then used to predict the functional genomic composition of the samples, and functional information is retrieved from the Integrated Microbiome Genomes database (33). Additionally, differences in components and KEGG metabolic pathways (34) are analyzed to characterize the differential functional genes in the microbial community’s metabolic pathways.

## DISCUSSION

Improving egg production is crucial for the poultry industry, especially in chicken farming, due to its significant economic benefits. Enhancing the egg-laying performance of chickens can substantially increase the total egg production of the flock, reduce the cost of egg production, and enhance the economic benefits for farmers (35). As the population grows and consumption habits change, the demand for eggs continues to rise. (36) Increasing egg production can better meet the market demand for eggs (37). Improving the egg-laying performance of chickens is becoming increasingly important (38).

Studying the composition and structure of the chicken gut microbiome can provide a better understanding of the unique gut microbial community specific to chickens, which in turn can enhance egg production performance through the modulation of the gut microbiota (39). With the advancements in 16S rRNA gene amplicon sequencing technology, further exploration has been conducted on the relationship between the gut microbiota and chickens with different egg production capabilities, such as studies on the relationship between gut bacteria and laying capacity (40).

The gut microbial community is composed of various types of microorganisms whose interactions significantly affect the host (41). In this study, significant differences in fecal microbial diversity and abundance were observed among three groups of chickens categorized by egg production level–low, medium, and high (p<0.05). The alpha diversity of the fecal microbiota in low and medium egg production chickens was significantly higher than in high egg production chickens. For example, Wang et al. found that analyzing the gut microbiota of broilers with high or low egg production performance revealed that adding *Enterococcus* to the diet can improve egg weight (42). Han et al. found significant differences in microbial diversity and community composition between high and low egg-producing Taihang chickens (43), consistent with our results. From the perspective of β-diversity, the PCoA plot shows significant differences between the high egg production group and other groups. This is consistent with the findings of Wang and others, who observed significant differences in the composition of the gut microbiota among different groups of laying hens (44).

At the phylum level, *Firmicutes*, *Bacteroidetes*, *Actinobacteria*, and *Proteobacteria* are dominant, which is consistent with the findings of Xiao and others. Their research indicated that *Firmicutes*, *Bacteroidetes*, *Proteobacteria*, *Actinobacteria*, and Cyanobacteria are the main microbial groups, with *Firmicutes* being the dominant phylum in the duodenum, jejunum, ileum, and colon (45). In this study, it was found that the proportions of *Firmicutes* and *Proteobacteria* in the gut of high-egg production chickens are higher than those in low- and medium-production chickens. Research has shown that the *Firmicutes* phylum in the gut possesses many genes responsible for fermenting dietary fibers and can also interact with the intestinal mucosa (46) Dietary fiber is an important nutrient that can influence the egg production performance of chickens (47). For example, lignocellulose is a useful source of dietary fiber, and its positive impact on the growth and egg production performance of chickens has been demonstrated (48), The presence of a higher content of *Firmicutes* may be one of the reasons for the high egg production in chickens. Furthermore, variations in the fecal microbial community, such as changes in the *Firmicutes/Bacteroidetes* ratio, significantly impact the different egg production levels of chickens. It has been demonstrated that the *Firmicutes/Bacteroidetes* ratio plays a crucial role in indicating the state of intestinal bacteria, and an increase in this ratio is closely associated with improved egg production performance (49). Additionally, as the dominant beneficial microbial communities in the chicken gut, both *Firmicutes* and *Bacteroidetes* are associated with the metabolism of short-chain fatty acids (50). Short-chain fatty acids are an important energy source for intestinal cells, promoting cell proliferation and regeneration, and have a positive effect on the egg production performance of chickens (51).

Additionally, the core fecal microbiome can moderately reflect the microbial structure in the gut at the species-specific level of microbial populations (52). Lactobacillus is often considered a beneficial bacterium in the gut (53). The high-egg production group has the highest levels of Lactobacillus and *Enterococcus* genera, and some studies have shown that Lactobacillus has a positive effect on improving gut health and production performance (54). Research has demonstrated that adding 0.6% of metabolic products containing Lactobacillus strains to the diet of laying hens can promote an increase in egg production rate (55). Additionally, supplementing the diet of laying hens with *Enterococcus* faecium also significantly increases the egg production rate (56). The levels of these genera are higher in the high-egg production group compared with the low- and medium-egg production groups. The aforementioned points indicate that changes in the gut microbiota can influence the differences in egg production levels in chickens.

We further investigated the differences in significant microbial species in the gut microbiota of chickens with varying egg-laying performances, aiming to identify biological markers that could explain the variations in egg production. The results indicated that an unclassified species within the *Limosilactobacillus* family and an unclassified species within the Bacilli genus are the main biomarkers for high-egg production chickens. This finding provides a new perspective for understanding the role of microbiota in egg production performance (57). The presence of these biomarkers suggests that specific microbial communities may enhance egg production performance by influencing gut health or nutrient absorption efficiency in chickens (58). For example, studies have shown that the *Limosilactobacillus* family can indirectly improve the egg production performance of chickens (59). Bacilli is a common class of bacteria, and some Bacillus species can be used as probiotics. These bacteria are present in the digestive tract of chickens and may also be found in the chicken house environment, thereby affecting egg production levels and health. For example, research by Jayaraman S. and others has shown that Bacillus subtilis PB6 can prevent necrotic enteritis and improve poultry production performance (60).

Microbes have different functions and relationships with their hosts (61). In the low-egg production group, chickens are primarily involved in amino acid metabolism, energy metabolism, folding, sorting and degradation, nucleotide metabolism, and translation. Ricke S. C. and others studied the factors affecting the gut microbiota in laying hens. They found that lactic acid bacteria break down feed proteins into peptides and amino acids, improving nutrition and health. Additionally, *Bifidobacteria* enhance energy metabolism by fermenting indigestible carbohydrates into short-chain fatty acids (62). In the metabolism of amino acids, Taurine is a sulfur-containing amino acid. Research by Li and others has found that the gut microbiota is involved in regulating inflammatory pathways. The taurine produced in this process is a potent antioxidant that can protect cells from damage caused by free radicals (63). This is particularly important for rapidly growing poultry, as antioxidants can enhance the growth performance and reproductive efficiency of production animals (64). In addition, the gut microbiota of chickens with high egg production primarily participates in the metabolic activities of cofactors and vitamins. Research by Lei and others has found that *lactobacilli* are crucial in the synthesis of B-group vitamins. These vitamins act as cofactors in various metabolic processes, regulating enzyme activity, which in turn affects egg production performance (65). Additionally, certain vitamins, such as vitamin E (66), have significant impacts on embryonic development. Providing sufficient levels of certain vitamins can promote the normal development of avian embryos, thereby increasing hatchability (67).

In ongoing fecal microbiota functional predictions, Pascal Andreu and colleagues applied the gut SMASH algorithm to systematically analyze the gut microbiome metabolism of 1,135 individuals from a Dutch cohort. They discovered significant differences in the distribution of inter-pathway connections, indicating that pathway-specific gene regulation and metabolite flux play crucial roles (68). Zhao and others used 16S rRNA and metagenomics to analyze the cecal microbiota in broilers and laying hens, finding that *Enterococcus faecium* as a probiotic improves intestinal integrity and reduces pathogen colonization, such as *Salmonella* (69). El-Sayed M. Abdel-Kafy and colleagues have found that dietary interventions can alter the composition and function of the microbiota. For instance, supplementing laying hen diets with xylanase does not significantly change overall production performance, but it can improve specific egg quality parameters, such as shell thickness and internal egg quality. Additionally, the supplementation of xylanase is associated with an increase in the number of lactobacilli in the feces, indicating positive regulation of the gut microbiota composition (70). These studies demonstrate that gut microbiota play specific roles in poultry production, such as managing the intestinal microbiome through specific interventions like probiotics and enzymes. These interventions impact the gut health and production performance of chickens, highlighting the importance of targeted microbial management in enhancing overall poultry productivity.

### Conclusion

This study analyzed the gut microbiota composition of chickens with different egg production capabilities, showing that the gut microbiota is significantly associated with egg production levels. Based on 16S rRNA amplicon sequencing results, chickens in the high-egg production group had higher abundances of the phylum *Firmicutes* and the genus *Lactobacillus*, which correlated with increased egg production. Additionally, significant differences in gut microbiota composition were observed among chickens with varying egg production levels. The high-egg production group compositions also exhibited a high abundance of *Enterococcus*, which may contribute to improve intestinal health and production performance. Furthermore, the low- and medium-egg production groups showed higher α-diversity indices compared with the high-production group, suggesting a relationship between gut microbial diversity and egg production. This study provides valuable insights into the gut microbiota of chickens with varying egg production capabilities and offers guidance for future strategies to improve egg production performance.

## Data Availability

The raw sequence data used in this manuscript have been deposited in the Genome Sequence Archive in National Genomics Data Center under accession number PRJCA031823.

## SUPPLEMENTAL MATERIAL

Fig. S1 spectrum.01927-24-s0001.pdf Sequencing data overview.

Fig. S2 spectrum.01927-24-s0002.pdf Comparison of fecal microbial α-diversity indices among three groups of chickens with different egg production.

Fig. S3 spectrum.01927-24-s0003.pdf Analysis of β-diversity indices for chickens with different egg production levels.

Fig. S4 spectrum.01927-24-s0004.pdf Differences in intestinal microbial abundance among chickens with different egg production levels.

Fig. S5 spectrum.01927-24-s0005.pdf LEfSe analysis of intestinal microbiota in chickens with different egg production levels, among bacterial species.

Fig. S6 spectrum.01927-24-s0006.pdf LEfSe analysis of intestinal microbiota in chickens with different egg production levels, among breeds.

Fig. S7 spectrum.01927-24-s0007.pdf Differential analysis of Level 3 KEGG metabolic pathways.

Supplemental legends spectrum.01927-24-s0008.docx Legends for Fig. S1 to S7.

Table S1 spectrum.01927-24-s0009.xlsx Differences of intestinal flora in the first 10 phyla of different laying hens.

Table S2 spectrum.01927-24-s0010.xlsx Differences of intestinal flora of the top 10 genera in different laying hens.

ASM does not own the copyrights to Supplemental Material that may be linked to, or accessed through, an article. The authors have granted ASM a non-exclusive, world-wide license to publish the Supplemental Material files. Please contact the corresponding author directly for reuse.
