# Supplementary figures and images for: Impact of gut microbial diversity on egg production performance in chickens
Source: Microbiol Spectr. 2025 Jan 14;13(2):e01927-24. doi: 10.1128/spectrum.01927-24 (PMC11792489; doi:10.1128/spectrum.01927-24)

**A**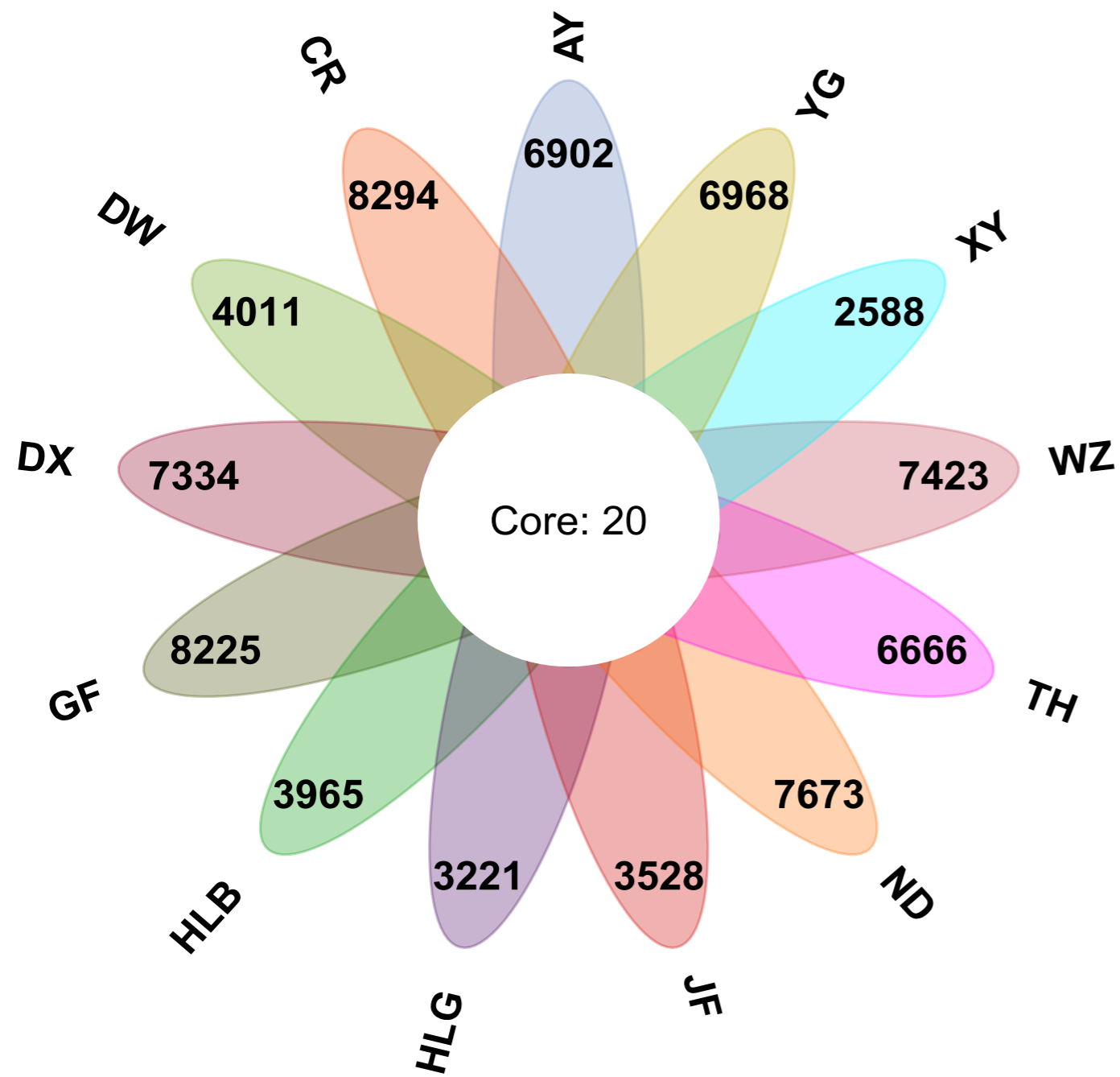**B**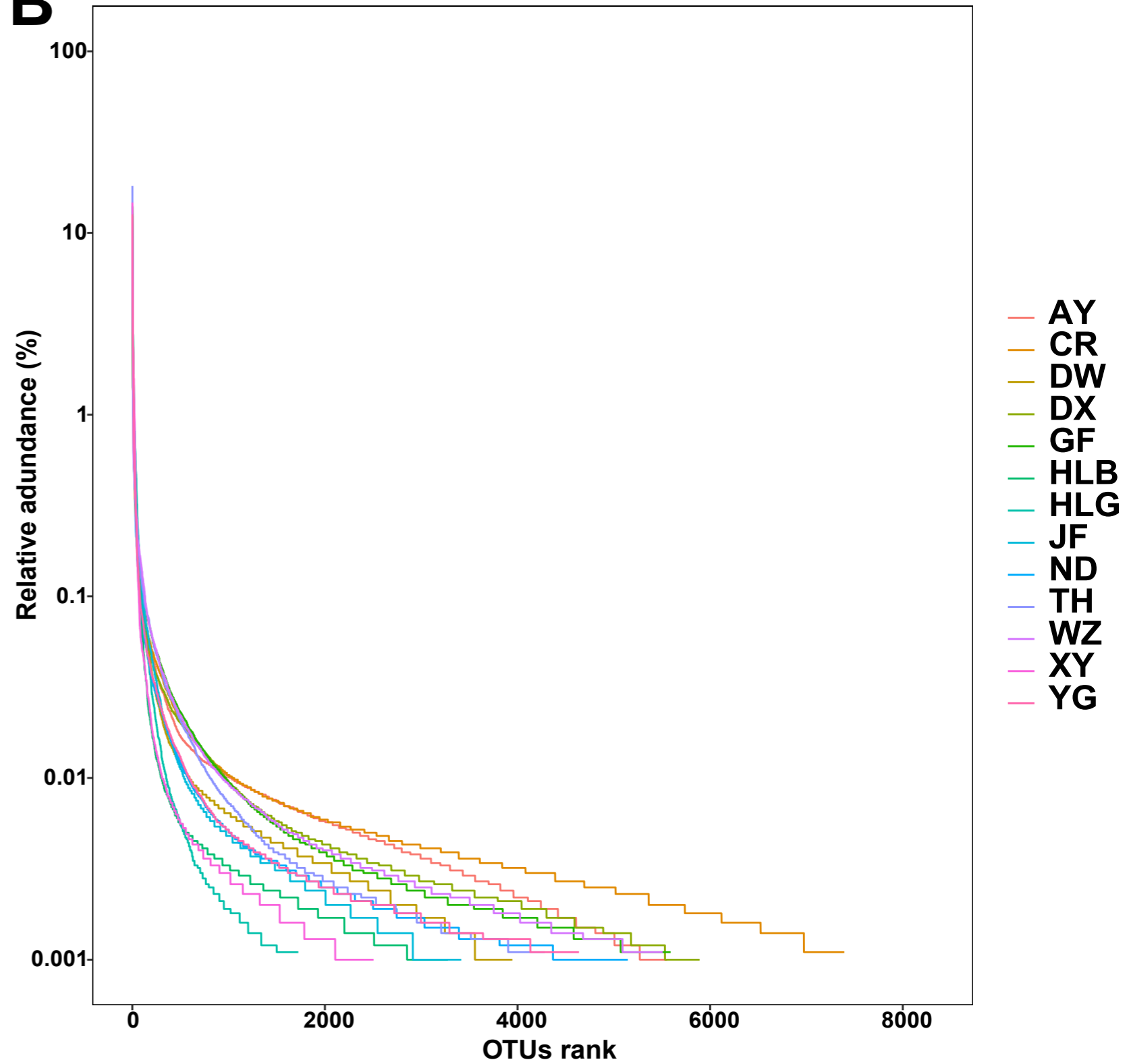

Supplement: Fig. S1 — Sequencing data overview. [file spectrum.01927-24-s0001.pdf]

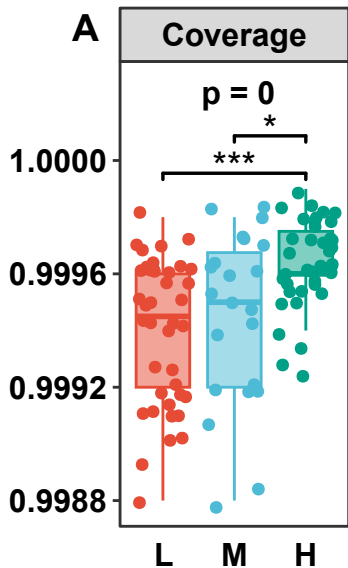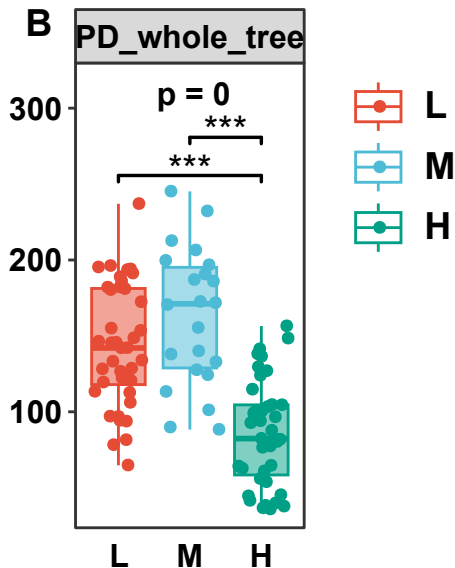

Supplement: Fig. S2 — Comparison of fecal microbial α-diversity indices among three groups of chickens with different egg production. [file spectrum.01927-24-s0002.pdf]

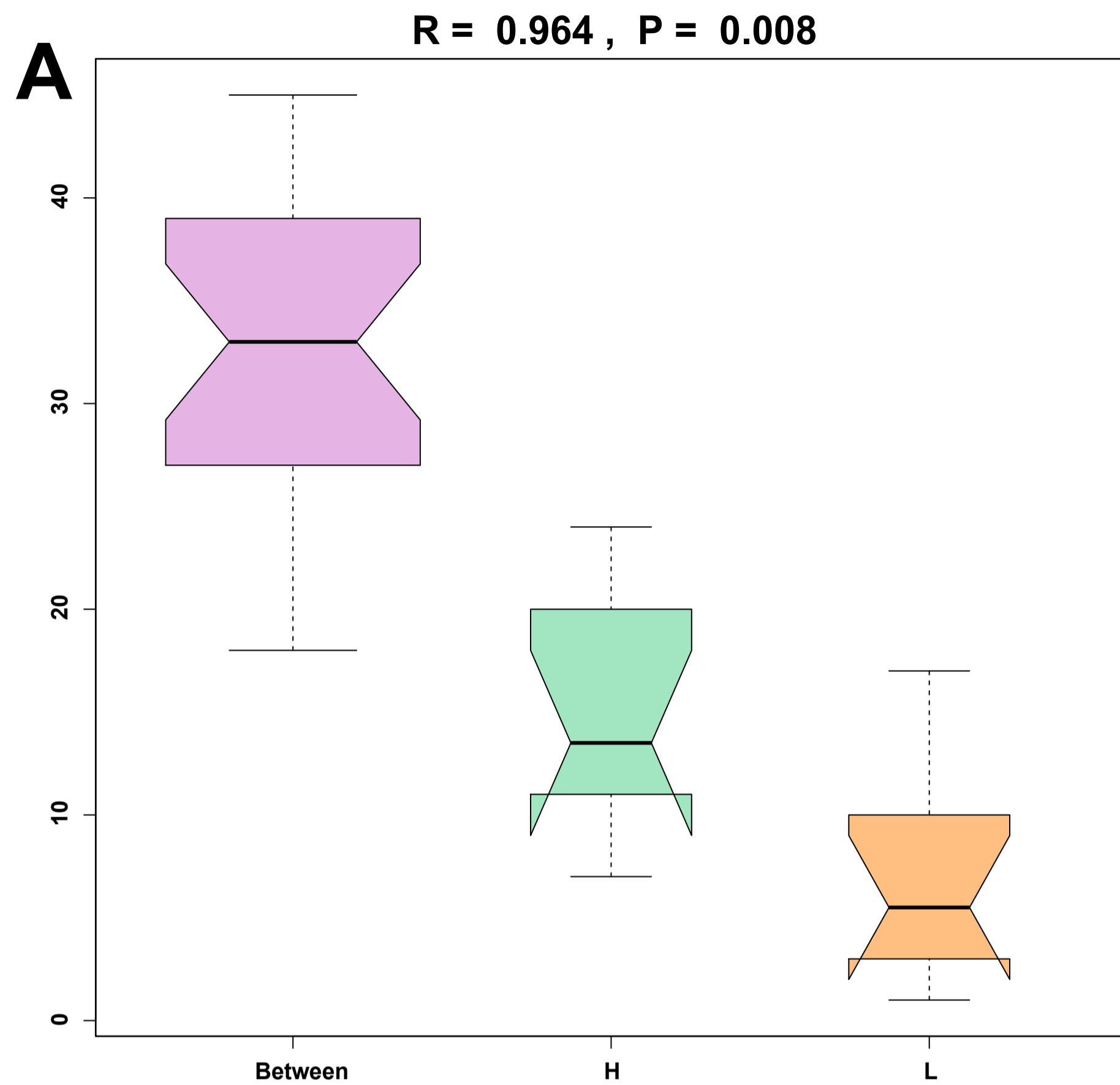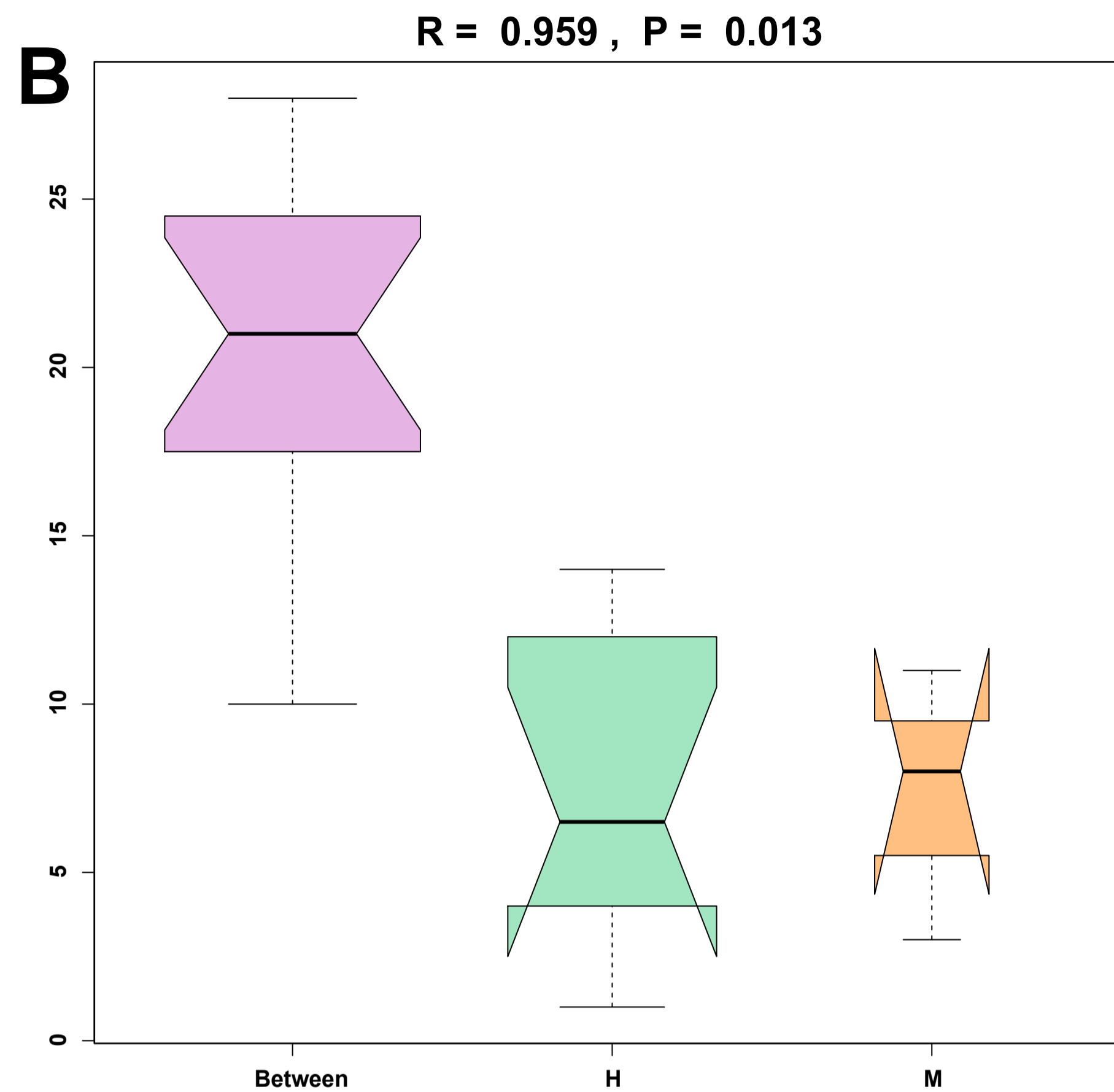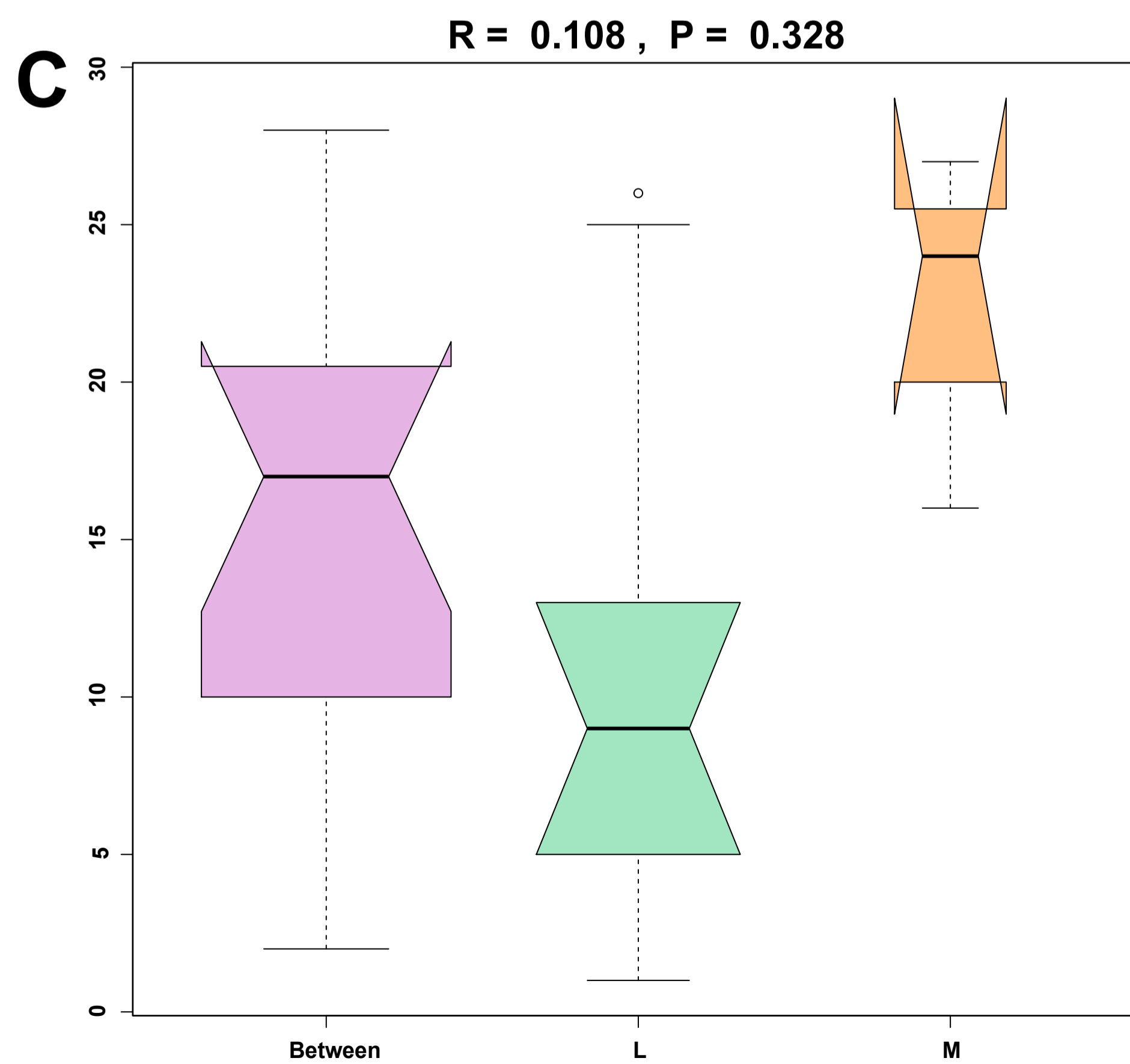

Supplement: Fig. S3 — Analysis of β-diversity indices for chickens with different egg production levels. [file spectrum.01927-24-s0003.pdf]

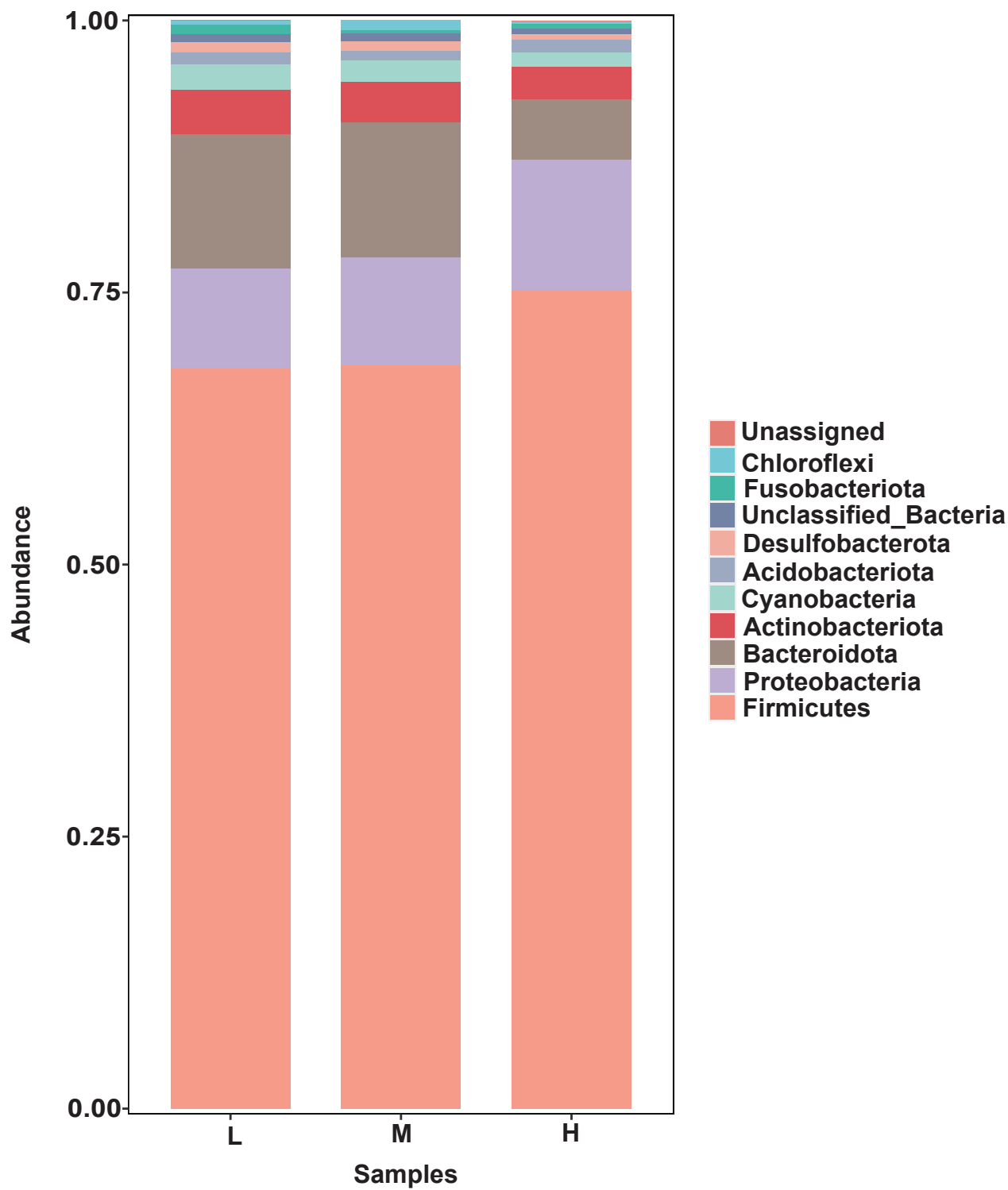

Supplement: Fig. S4 — Differences in intestinal microbial abundance among chickens with different egg production levels. [file spectrum.01927-24-s0004.pdf]

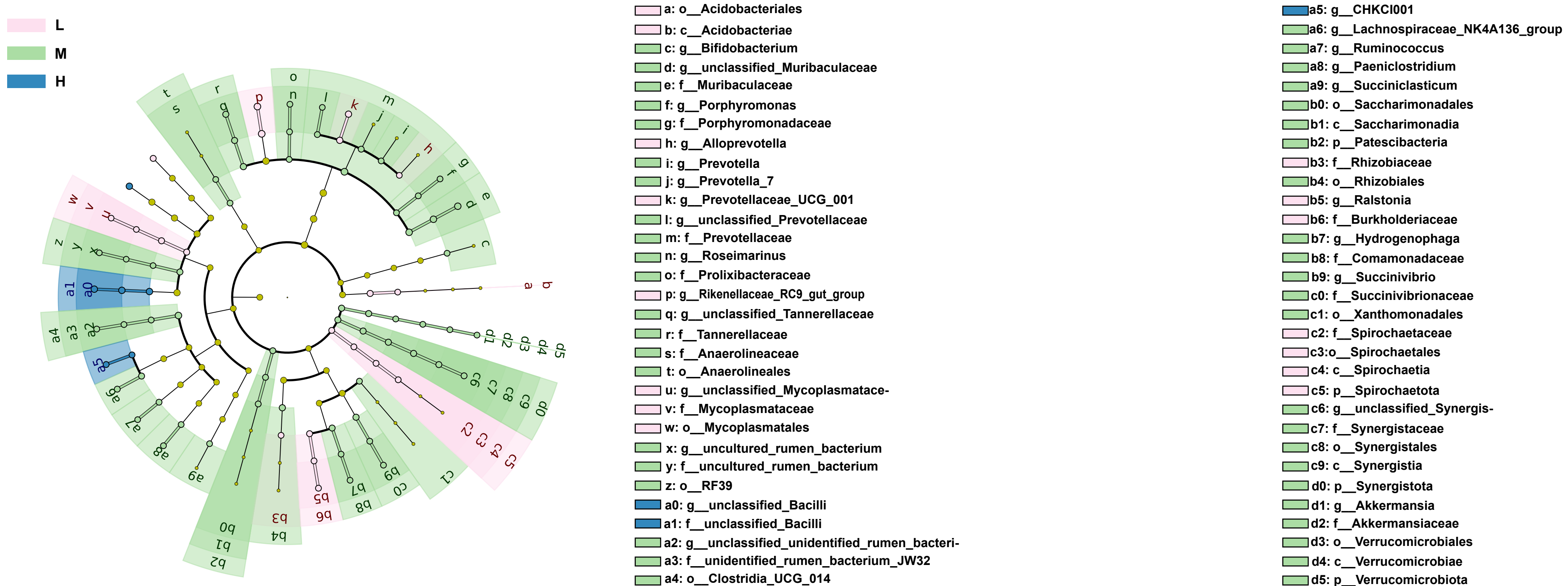

Supplement: Fig. S5 — LEfSe analysis of intestinal microbiota in chickens with different egg production levels, among bacterial species. [file spectrum.01927-24-s0005.pdf]

L M H

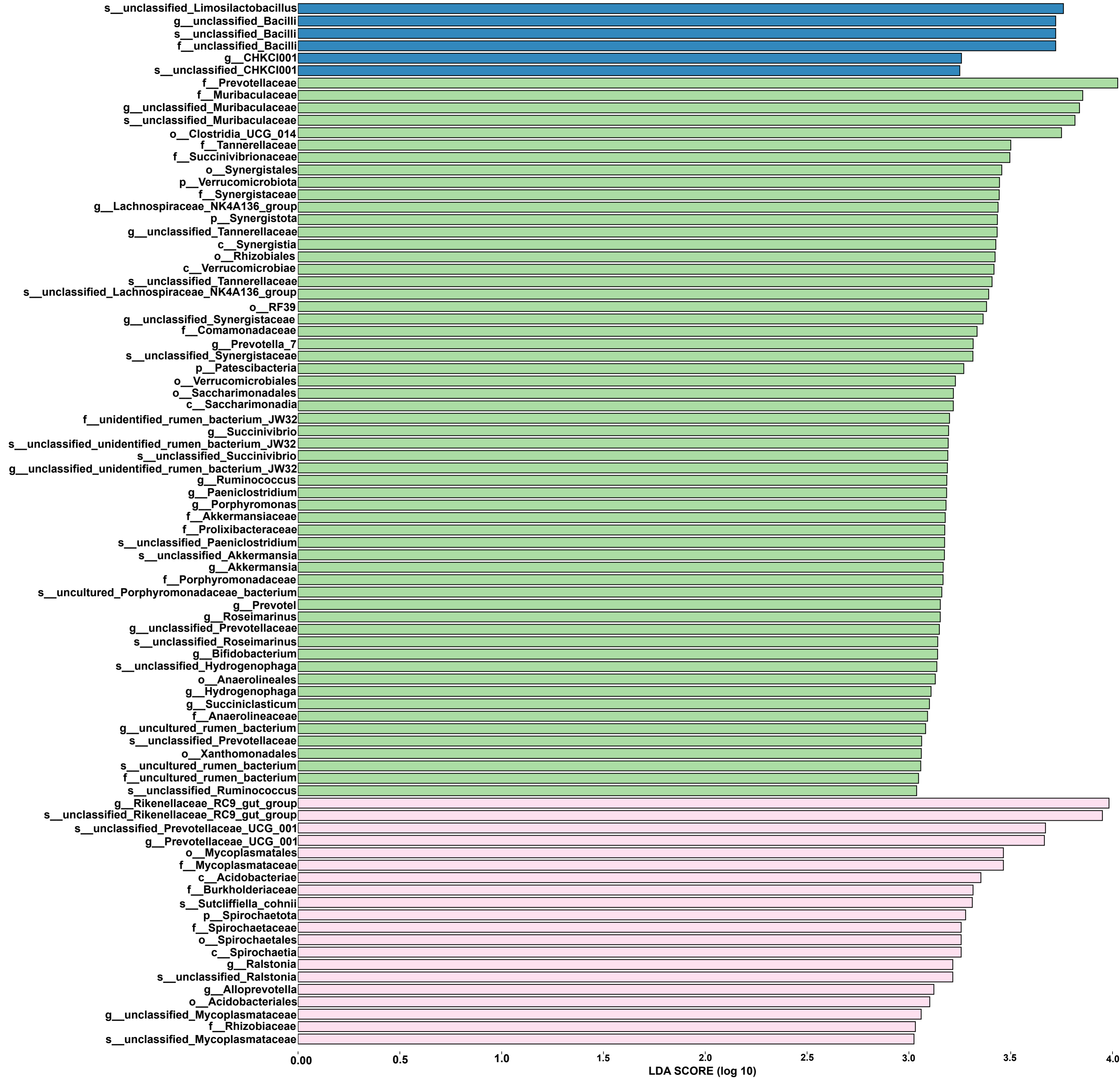

Supplement: Fig. S6 — LEfSe analysis of intestinal microbiota in chickens with different egg production levels, among breeds. [file spectrum.01927-24-s0006.pdf]

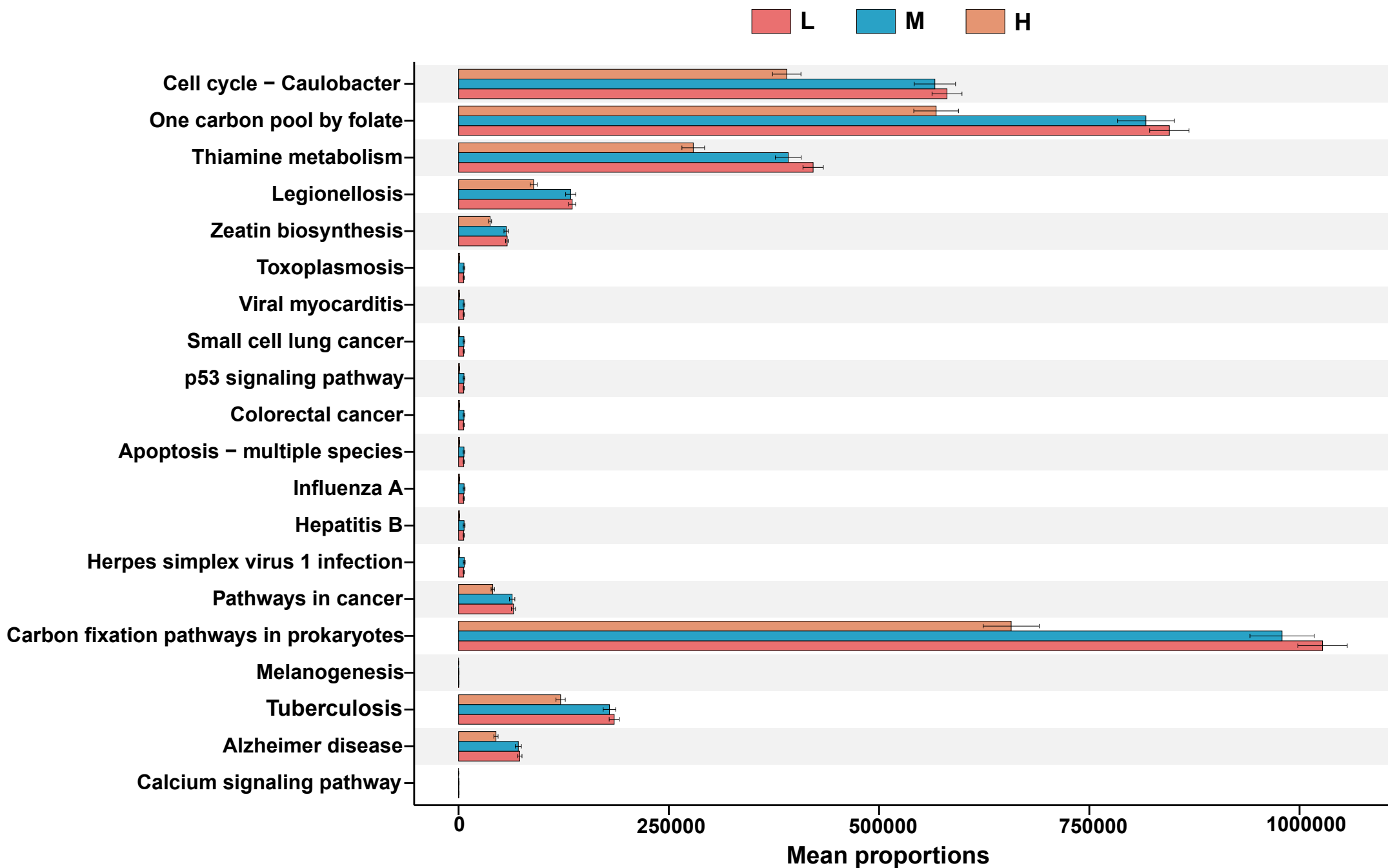

Supplement: Fig. S7 — Differential analysis of Level 3 KEGG metabolic pathways. [file spectrum.01927-24-s0007.pdf]
