# Supplementary material for: Impact of gut microbial diversity on egg production performance in chickens
Source: Microbiol Spectr. 2025 Jan 14;13(2):e01927-24. doi: 10.1128/spectrum.01927-24 (PMC11792489; doi:10.1128/spectrum.01927-24)
Supplement: Supplemental legends — Legends for Fig. S1 to S7. [file spectrum.01927-24-s0008.docx]

**Supplementary Information**

**Additional file 1: Table.S1** Differences of intestinal flora in the first 10 phylum of different laying hens.

**Table.S2** The difference of intestinal flora of the top 10 genus in different laying hens.

**Fig.S1** **Sequencing data overview supplement**:(A) OTU petal diagrams for different breeds (B) Rank abundance curves for different breeds. Sequencing data overview supplement.

**Fig.S2** **Comparison of fecal microbial α-diversity indices among three groups of chickens with different egg production**: (A) (B) α-diversity analysis of the microbial communities in the three groups based on microbial diversity indices (Coverage and PD_whole_tree).

**Fig.S3 Analysis of β-diversity indices for chickens with different egg production levels**: Variance analysis of pairwise comparisons between two groups of chickens with different egg productions.

**Fig.S4 Differences in intestinal microbial abundance among chickens with different egg production levels**: Differences in the abundance of the top 10 phyla of intestinal microbiota among chickens with varying egg production levels.

**Fig.S5 LEfSe analysis of intestinal microbiota in chickens with different egg production levels**: phylogenetic trees of bacterial species at various taxonomic levels with different abundances and an LDA score > 3.

**Fig.S6 LEfSe analysis of intestinal microbiota in chickens with different egg production levels**: LEfSe analysis among different breeds, identifying biomarkers with an LDA threshold ≥ 3.

**Fig.S7 Differential analysis of Level 3 KEGG metabolic pathways**: functional differences among three groups with varying egg production levels.
